# Radiation Induced Abscopal Effect in a Patient With Malignant Pleural Mesothelioma on Pembrolizumab

**DOI:** 10.7759/cureus.22159

**Published:** 2022-02-12

**Authors:** Rebekah Rittberg, Elisa Chan, Stephen Yip, Deepu Alex, Cheryl Ho

**Affiliations:** 1 Medicine, University of British Columbia, Vancouver, CAN; 2 Medical Oncology, BC Cancer, Vancouver, CAN; 3 Radiation Oncology, BC Cancer, Vancouver, CAN; 4 Pathology, BC Cancer, Vancouver , CAN; 5 Pathology, BC Cancer, Vancouver, CAN

**Keywords:** pembrolizumab, immune modulation, radiotherapy, mesothelioma, immunotherapy, abscopal effect

## Abstract

Abscopal effect is a rare phenomenon in which treatment benefit from radiotherapy (RT) is seen outside the target field due to activation of the immune system inducing an anti-tumor effect. This phenomenon has been reported in cancer patients receiving immune checkpoint inhibitors (ICI). Here we report a case of presumed abscopal effect in malignant mesothelioma. The patient received second-line single-agent pembrolizumab however had disease progression after four cycles leading to palliative RT (20 Gray) to the right mainstem bronchus. Follow-up radiographic imaging confirmed benefit and pembrolizumab was continued. Follow-up computed tomography (CT) five months after RT, showed marked radiographic improvement of all measurable diseases with improvement in right-sided aerated lung volume. Because of the original disease progression on pembrolizumab, with marked improvements within and outside the RT field after RT, treatment response was presumed due to the abscopal effect.

## Introduction

The inflammatory response to asbestos fibers contributes to the development of malignant mesothelioma leading to an immunosuppressed microenvironment that dampens antitumor immune function [1]. Immune checkpoint inhibitors (ICI), which result in modulation of the tumor microenvironment, have been of interest and found to improve outcomes in mesothelioma [2].

Radiotherapy (RT) is also a highly effective and integral component of cancer treatment in countless malignancies. RT uses high-energy particles to produce direct damage to deoxyribonucleic acid (DNA) and indirect damage through the production of free radicals resulting in apoptosis of cells within the target region [3]. An uncommon phenomenon has been observed when recipients of RT display a response inside and outside the targeted region [4]. This became known as the abscopal effect and is believed to occur due to RT changing the tumor microenvironment leading to increased antigen expression resulting in tumor-specific immunity [3]. The abscopal effect has been rarely reported but, over the last decade, has received increasing attention with the use of ICI with immune activation [5]. The abscopal effect has limited reporting in mesothelioma and is more commonly seen in melanoma and non-small cell lung cancer. Here we present a case of the presumed abscopal effect in malignant mesothelioma in a patient treated with pembrolizumab.

## Case presentation

**Figure 1:**
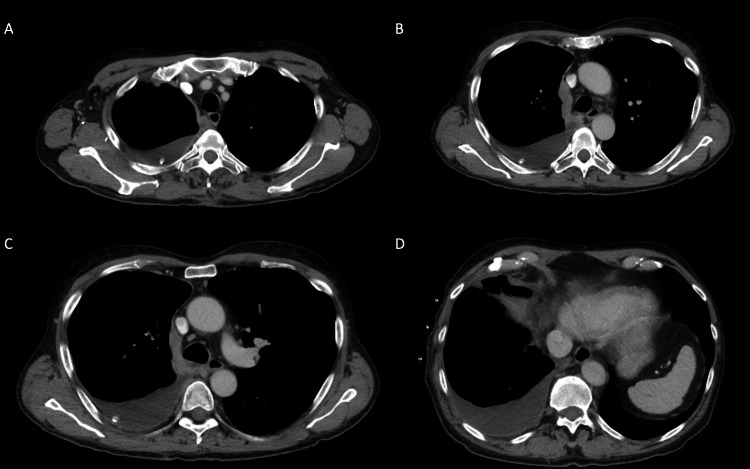
CT Chest at time of diagnosis, after pleural effusion drainage. A) Pleural-based disease along superior aspect of mediastinum measuring thickness of 12 mm. B) Pleural-based conglomerate abutting lateral superior mediastinum, measuring up to 17 mm in thickness. C) Pleural-based plaque extending down mediastinum. D) Pleural effusion at right lung base.

A 68-year-old male, with a history of asbestos exposure, presented with four months of exertional dyspnea, was found to have a massive right-sided pleural effusion with a complete collapse of the right lung. After five failed attempts for cytology from thoracentesis, a pleural biopsy was completed with pathology confirming epithelial malignant pleural mesothelioma (Figure 1). Immunohistochemistry (IHC) phenotype: tumor cell stained strongly positive for calretinin, cytokeratin-5 (CK5), and Wilms' tumor 1 (WT-1) and negative for claudin-4. Programmed death ligand 1 (PD-L1) tumor proportion score was 1-5% using the Dako PD-L1 IHC 22C3 pharmaDx kit. Next-generation DNA/RNA (ribonucleic acid) panel-based mutation screen was negative. Due to the extent and location of the pleural-based plaques, the disease was deemed inoperable.

Due to minimal symptoms after insertion of the indwelling pleural catheter, and excellent performance status, the patient wished to be surveilled as opposed to starting palliative systemic therapy. He was surveilled for nine months before clinically and radiographically demonstrating disease progression. At that time computed tomography (CT) demonstrated lobulated soft tissue masses within the right pleural space. Thickness at the level of the aortic arch was 61 mm and at the carina was 24 mm. The right chest wall muscle metastasis measured 35x89 mm. He was started on carboplatin (AUC 5) and pemetrexed (500 mg/m2) with cycle five complicated by a Pseudomonas aeruginosa indwelling pleural catheter infection requiring its removal. CT after completion of chemotherapy demonstrated reduced tumor size with pleural thickness at the level of the aortic arch now measuring 16 mm and 10 mm at the carina. The right chest wall metastasis measured 35x77 mm. 

**Figure 2:**
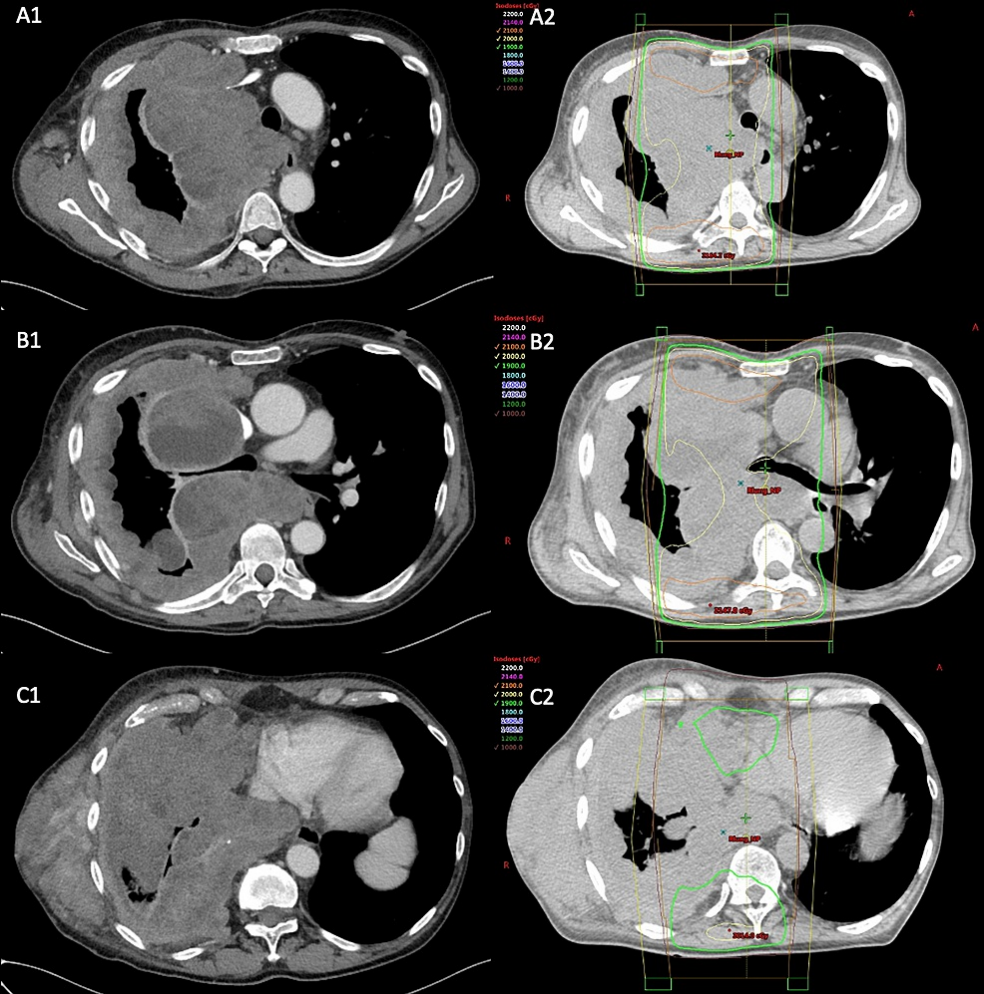
CT Chest prior to RT, after 4 cycles of pembrolizumab. Approximated aerated right lung is 15%. A1) Pleural-based conglomerate abutting the lateral superior mediastinum measuring up to 85x152 mm. B1) Tumor compression of right mainstem bronchus. C1) Lateral chest wall mass, between right lateral latissimus dorsi, and lateral intercostal/rib measures 75x146 mm. Infiltrative tumor involving inferior aspect of right lung. A2, B2, and C3 are corresponding RT plan illustrating extent of disease outside of the field.

Treatment was held as there was disease stability. Six months after completion of chemotherapy there was radiologic evidence of disease progression with the right chest wall metastasis now measuring 72x55 mm with further pleural thickening. He was started on second-line pembrolizumab 200 mg flat dosing administered every 21 days. Throughout the first four cycles, there was concern about clinical and radiographic progression (Figure 2).

**Figure 3:**
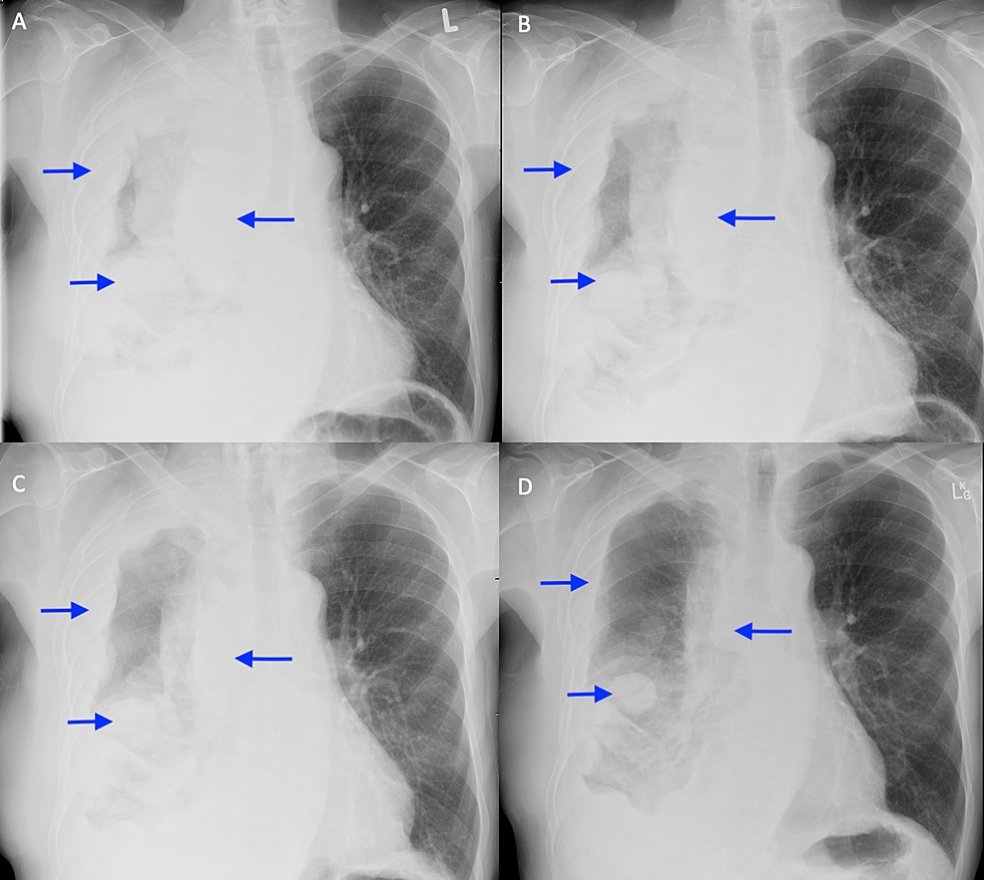
Chest x-rays after RT completed illustrating disease response. A) Radiograph taken on last day of RT. B) 3 weeks after RT completed. C) 9 weeks after RT completed. D) 14 weeks after RT completed.

**Figure 4:**
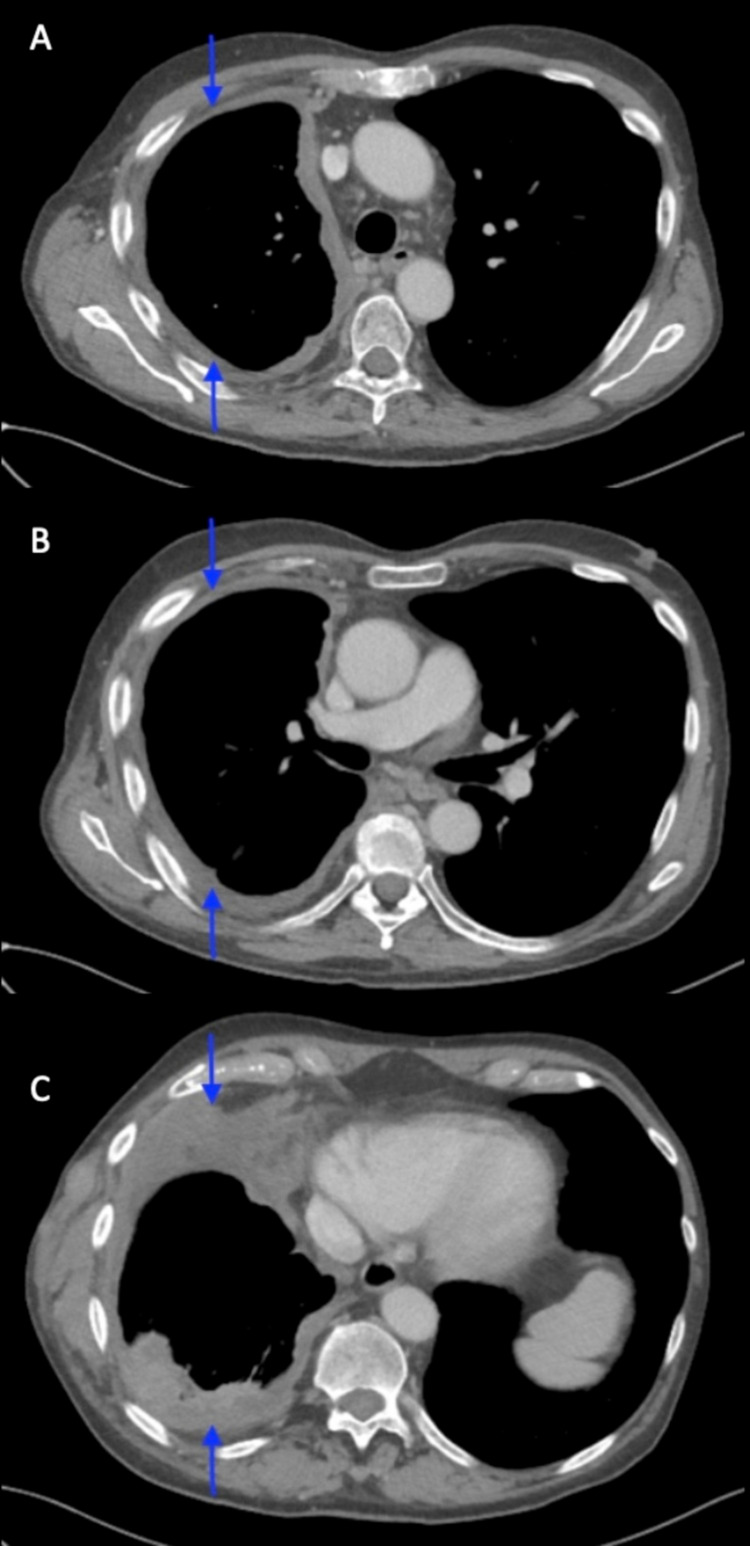
CT Chest 5 months after completion of RT, after 12 cycles of pembrolizumab. Approximated aerated right lung is 90%. A) Right pleural based confluent mass has substantially improved to 12x1mm along the lateral superior mediastinum. B) Compression of right mainstem bronchus resolved. C) Lateral chest wall mass decreased to 23x80mm. Reduction in extent of tumor mass.

Due to progressive dyspnea and clinical signs of progression, he received palliative RT, 20 Gray in five fractions, to the right mediastinal pleura as there was compressing of the right mainstem bronchus. RT was administered six weeks after cycle four infusion was received. He was planned to switch to chemotherapy however RT began to improve his dyspnea and therefore pembrolizumab was continued with cycle five administered three days after RT was completed. Follow-up chest x-rays illustrated response (Figure 3) and CT Chest was completed five months after RT was concluded demonstrated profound radiographic improvement, including regions well outside of the radiation field (Figure 4). 

Pembrolizumab was continued for a total of 13 cycles. Unfortunately, the patient then had a rapid progression of dyspnea, with worsening pleural effusion and lung infiltration. Pneumonitis was ruled out. His clinical status rapidly deteriorated and the patient passed away 43 months after his original diagnosis of unresectable malignant mesothelioma. 

## Discussion

This case report illustrates the presumed abscopal effect in malignant mesothelioma. Although the delayed response to pembrolizumab cannot be entirely excluded, clinical response after progression of four cycles is extremely unlikely especially after clinical and radiographic signs of disease progression. This case report adds to the limited evidence of abscopal effect in malignant mesothelioma and minimal body of literature within other malignancies [5]. With the relative rarity of mesothelioma and the newer introduction of ICI, only two cases have previously been published demonstrating an abscopal effect in malignant mesothelioma. Barsky et al. reported on a 67-year-old male with epithelioid pleural mesothelioma who received interferon-alpha gene therapy, as part of a clinical trial. During this treatment, he had palliative RT (30 gray in 10 fractions) for bulky right hemithorax disease with the subsequent CT imaging demonstrating dramatic response, two months after RT, both within and outside of the RT field [6]. Mampuya et al. reported on a 72-year-old male who had progressive disease on chemotherapy and was started on second-line pembrolizumab. After 20 cycles he had progressive disease leading to administration of palliative RT (30 gray in 10 fractions) to the paramediastinal lesion. Imaging three months after RT demonstrated partial response to paramediastinal lesion as well as response to right pleura lesion, outside the RT field [7]. 

Historically, it was believed that RT only impacted tissue within the targeted field however increasing clinical and experimental data demonstrates that RT also impacts the tumor microenvironment. Although the exact mechanism of the abscopal effect is still not known, it is hypothesized that the release of tumor cell neoantigens activates tumor-specific T-cells allowing for immunogenic cell death outside of the irradiated region [3]. Since the first case of an abscopal effect was reported by Mole et al. in 1953 [4], only 46 cases were reported between 1969 and 2014, despite millions of patients receiving RT. During this period the largest proportion of cases were reported in renal cell carcinoma, melanoma, and lymphoma, tumors conventionally considered immunogenic [5]. Since 2014, there has been the rapid introduction of ICIs in various tumor types, drastically changing the treatment algorithm for countless malignancies. With the increased use of ICI, an immune-activating treatment, there has been an increasing rate of published cases illustrating the abscopal effect.

A systematic review of published abscopal effect cases found a median RT dose required to induce an abscopal effect was 31 Gray (range from 0.45 to 74.8 Gray) and can occur regardless of fractionation or delivery modality [5, 8]. The median time to observe the abscopal effect was two months (range from 0 to 24 months) [5]. It is currently postulated that hypofractionated or stereotactic body RT may have added benefits as they result in higher rates of immunogenic cell death [9]. 

ICI has also changed the treatment algorithm in mesothelioma. ICI was first evaluated in the second-line setting with pembrolizumab in KEYNOTE-028, an anti-programmed death 1 (PD-1) antibody, found to have an overall response rate (ORR) of 20%, with an additional 52% having stable disease, in PD-L1 positive patients (1% or greater) [10]. However, in phase III randomized control trial, PROMISE-meso trial, pembrolizumab was compared to single-agent gemcitabine or vinorelbine. ORR was higher with pembrolizumab (22% versus 6%), however, neither progression-free survival (PFS) nor overall survival (OS) was improved [11]. Nivolumab, another anti-PD-1 antibody, was considered with and without ipilimumab, a cytotoxic T-lymphocyte-associated protein 4 (CLTA-4) antibody, with an ORR of 19% in the nivolumab arm and 28% in the nivolumab and ipilimumab arm. An exploratory analysis found that in PD-L1 positive tumors (1% or greater) ORR was 39% compared to 12% in PD-L1 negative tumors [12]. Preliminary results from CONFIRM, phase III randomized control trial, which considered nivolumab to best supportive care demonstrated improved PFS (3.0 versus 1.8 months) and improved OS (9.2 versus 6.6 months) [13]. Avelumab, an anti-PD-L1 antibody, had an overall ORR of 9%, with ORR of 19% in PD-L1 positive (5% or greater) and 7% in PD-L1 negative tumors [14]. DETERMINE considered tremelimumab, another CTLA-4 antibody, was not found to have improved OS compared to best supportive management [15].

In the first-line setting, CheckMate 743 demonstrated improved OS with nivolumab and ipilimumab compared to platinum plus pemetrexed (18.1 versus 14.1 months), without a difference in OS based on PD-L1 positivity. Survival was improved in both epithelioid (18.7 versus 16.5 months) and non-epithelioid (18.1 versus 8.8 months) histology [2]. With this, the use of nivolumab and ipilimumab was approved for first-line therapy for all histologic subtypes. Although not currently known, the increased use of ICI in mesothelioma may lead to increasing number of reported cases of abscopal effect. 

Biomarkers to predict ICI response are still needed in mesothelioma. PD-L1 expression may be measured however are positive in 20-70% of patients. The wide range may be due to histologic variance between cohorts, variable threshold of positivity and differing antibodies used [16]. Tumor mutation burden has also been evaluated and felt not to correlate with immune checkpoint inhibitor response. Ultimately, in malignant mesothelioma, there is a need to identify new biomarkers, and broad-based combined DNA and RNA panels should be completed, when possible, to search for unexpected mutations although commonly negative, as found in our patient [17]. 

There is an increasing consensus that combining RT with ICI may create an opportunity to intentionally induce an abscopal effect resulting in improved local and distant disease control with lower total doses of RT reducing toxicity. Ongoing clinical trials are evaluating the inducibility of an abscopal effect in patients receiving RT and ICI in non-small cell lung cancer (NCT04245514, NCT04530708), pancreatic cancer (NCT04212026), and hepatocellular carcinoma (NCT03316872). 

## Conclusions

Malignant mesothelioma continues to be a highly morbid malignancy requiring ongoing research and exploration into treatment optimization. Here we present the third case of the presumed abscopal effect in malignant mesothelioma due to RT in combination with ICI. Added immune activation with RT is a hypothetical mechanism to optimize treatment response but many unanswered questions remain including optimal RT dose, fractionation, and timing with ICI.
